# A predictive model integrating iron metabolism and oxidative stress biomarkers with clinicopathological factors for recurrence and metastasis in oral squamous cell carcinoma

**DOI:** 10.3389/fonc.2026.1821007

**Published:** 2026-04-24

**Authors:** Jingyu Qi, Ke Liu, Yongwu Wang, Yuanqing Huang, Min Gan

**Affiliations:** Department of Stomatology, Affiliated Hangzhou First People’s Hospital, School of Medicine, Westlake University, Hangzhou, Zhejiang, China

**Keywords:** clinicopathology, iron metabolism, oral squamous cell carcinoma, oxidative stress, recurrence and metastasis

## Abstract

**Objective:**

To investigate the impact of iron metabolism and oxidative stress on recurrence and metastasis in oral squamous cell carcinoma (OSCC) and to develop an integrated predictive model.

**Methods:**

A retrospective study was conducted on 240 OSCC patients who underwent radical surgery. Preoperative iron metabolism indicators [serum iron (Fe), ferritin, transferrin (TF), total iron-binding capacity (TIBC), transferrin saturation (TSAT)] and oxidative stress indicators [malondialdehyde (MDA), reactive oxygen species (ROS), superoxide dismutase (SOD), glutathione peroxidase (GSH-Px), total antioxidant capacity (T-AOC)] were collected alongside clinicopathological data. Multivariable Cox regression was used to identify predictors, and a nomogram was constructed. Model performance was assessed via C-index, time-dependent ROC curves, and calibration plots.

**Results:**

The recurrence/metastasis rate was 36.7% (88/240). The recurrence/metastasis group showed higher proportions of T3-T4 stage, N+ status, poor differentiation, perineural invasion, and lymphovascular invasion (all *P* < 0.05). This group also displayed higher ferritin, MDA, and ROS levels, but lower Fe, TF, TIBC, TSAT, SOD, GSH-Px, and T-AOC levels (all *P* < 0.05). Ferritin was positively correlated with MDA, whereas Fe was positively correlated with SOD/GSH-Px and negatively correlated with MDA (all *P* < 0.05). T stage, N+ status, poor differentiation, elevated ferritin, and decreased TSAT, TF, and SOD were independent predictors (all *P* < 0.05). The nomogram showed excellent discrimination (C-index = 0.905) and high predictive accuracy (AUC: 0.948-0.979), with good calibration.

**Conclusion:**

Dysregulated iron metabolism and oxidative stress are independently associated with OSCC recurrence and metastasis. A model combining these serum biomarkers with traditional clinicopathological factors significantly improves risk stratification, offering a potential tool for personalized management.

## Introduction

Oral squamous cell carcinoma (OSCC) is one of the most common malignancies of the head and neck, characterized by high invasiveness, high local recurrence rates, and a propensity for cervical lymph node and distant metastasis (1–3). Despite continuous optimization of comprehensive treatment strategies, including surgical resection combined with radiotherapy, chemotherapy, targeted therapy, and immunotherapy, a considerable proportion of patients still experience recurrence or metastasis after radical treatment, leading to limited survival benefits (4–6). Clinical risk assessment primarily relies on pathological features such as TNM stage, tumor differentiation, margin status, perineural invasion (PNI), and lymphovascular invasion (LVI). However, significant prognostic heterogeneity persists among patients with the same stage or similar pathological features, suggesting that traditional indicators alone are insufficient to fully explain the biological heterogeneity of tumors (7–9). Therefore, identifying novel, accessible, and quantifiable biomarkers related to tumor progression and integrating them with clinicopathological factors to achieve more refined risk stratification is key to improving the prediction and management of OSCC recurrence and metastasis. Particularly in resource-limited clinical settings, early risk stratification based on preoperative serum biomarkers could guide more rational decisions regarding neck management, adjuvant therapy, and follow-up frequency.

Reprogramming of iron metabolism and imbalance in oxidative stress are crucial biological processes in tumor development and progression (10). Iron is essential for cell proliferation, DNA synthesis, and energy metabolism, but it can also catalyze the generation of reactive oxygen species (ROS) through the Fenton reaction, driving lipid peroxidation and oxidative damage. Tumor cells often exhibit iron dependence and alter iron homeostasis by regulating transferrin receptors, ferritin, and iron export pathways, thereby promoting invasion and metastasis or affecting treatment sensitivity (11, 12). Moreover, sustained oxidative stress can activate multiple pro-tumor signaling pathways, enhance epithelial-mesenchymal transition (EMT), matrix degradation, and angiogenesis, and interact with the remodeling of the immune microenvironment (13). Serum iron metabolism indicators such as ferritin, transferrin (TF) and transferrin saturation (TSAT), as well as oxidative stress and antioxidant indicators like malondialdehyde (MDA) and superoxide dismutase (SOD), are measurable under routine clinical laboratory conditions and hold translational potential. However, the integrated value of these indicators in assessing the risk of OSCC recurrence and metastasis remains insufficiently validated.

Therefore, this study intends to analyze, from the perspectives of iron metabolism and oxidative stress, the relationships of clinicopathological characteristics and preoperative serum indicators with recurrence and metastasis outcomes in OSCC patients undergoing radical surgery. The study aims to identify independent influencing factors and construct a predictive model to provide a basis for personalized risk stratification, follow-up strategy formulation, and exploration of potential intervention targets in OSCC.

## Materials and methods

### Sample size estimation

This was a single-center retrospective cohort study that aimed to construct a predictive model for recurrence and metastasis risk. The primary statistical method was multivariable logistic regression (outcome: occurrence of recurrence and/or metastasis). Sample size estimation followed the events per variable (EPV) principle commonly used in predictive model studies: a minimum of 10–15 outcome events per predictor variable is generally recommended to ensure stable regression coefficients and reduce overfitting risk (14). Considering the core predictors intended for the final model (e.g., T stage, N stage, differentiation grade, ferritin, TF, TSAT, SOD, totaling approximately 7 variables, with additional clinical covariates potentially included), a minimum of 70–105 recurrence/metastasis events would be required. Based on the actual enrollment of 240 patients with 88 recurrence/metastasis events, the EPV was approximately 12.6 (88/7), meeting the minimum requirement of EPV ≥ 10. Thus, the sample size provided basic statistical feasibility for conducting multivariable modeling and area under the curve (AUC) evaluation.

### Study population

A total of 240 patients with pathologically confirmed OSCC who underwent surgical treatment at our hospital between January 2017 and January 2025 were consecutively enrolled. Clinical, pathological, and laboratory data were retrospectively collected via the electronic medical record system (EMRS), and follow-up analysis was conducted.

Inclusion criteria were: confirmed OSCC diagnosis meeting pathological standards (15) and undergoing radical surgical resection; complete postoperative pathological data and serum test results with complete follow-up information; comprehensive clinical data including current/past medical history and personal/family history; and absence of concurrent tumors in other body sites. Exclusion criteria included: history of other malignancies; concurrent severe infectious or autoimmune diseases; incomplete follow-up data; preoperative treatment such as surgery, chemotherapy or radiotherapy; perioperative death; or inability to assess recurrence or metastasis outcomes. The study protocol was reviewed and approved by the Ethics Committee of our hospital.

### Collection of clinical data and clinicopathological characteristics

General clinical data, including gender, age, smoking history, alcohol consumption history, and comorbidities (e.g., hypertension, diabetes), were collected through a review of the EMRS. Tumor-related clinicopathological characteristics were systematically compiled, including primary tumor site, tumor size, T stage, regional lymph node status (N stage), pathological differentiation grade, PNI, LVI, margin status, presence of jawbone invasion, and treatment regimen. Tumor staging was performed according to the American Joint Committee on Cancer staging criteria (16).

### Detection of iron metabolism-related indicators

On the morning of surgery, fasting venous blood samples were collected from all patients. Samples were allowed to clot at room temperature for 30 minutes and then centrifuged at 3,500 rpm for 10 minutes to obtain serum. Iron metabolism-related indicators, including serum iron (Fe), ferritin, TF, total iron-binding capacity (TIBC), and TSAT, were measured using an automated biochemical analyzer (BK-200, BIOBASE, Jinan, Shandong, China). All assays were performed according to the manufacturer’s instructions, with internal quality control samples run in parallel to ensure accuracy and reproducibility. Measurements were completed within 2 hours of sample processing, and standard clinical laboratory procedures were strictly followed throughout.

### Detection of oxidative stress-related indicators

Oxidative stress status was assessed by measuring indicators related to lipid peroxidation and the antioxidant system. Lipid peroxidation was evaluated using MDA and ROS. Antioxidant defense capacity was assessed by SOD, glutathione peroxidase (GSH-Px), and total antioxidant capacity (T-AOC). On the morning of surgery, fasting venous blood samples were collected, allowed to clot at room temperature for 30 minutes, and centrifuged at 3,500 rpm for 10 minutes to obtain serum. All measurements were performed using commercially available kits (Nanjing Jiancheng Bioengineering Institute, Nanjing, China) according to the manufacturer’s instructions. Absorbance or fluorescence was read on an automated microplate reader (FC, Thermo Fisher Scientific, Shanghai, China). Internal quality control samples were included in each run to ensure assay reliability, and all measurements were completed within 2 hours of sample processing.

### Follow-up and outcome definition

Follow-up was conducted through outpatient review and telephone calls, starting from the date of surgical treatment completion. Follow-up frequency was every 3 months for the first 2 years, every 6 months from year 3 to year 5, and annually thereafter, with the follow-up cutoff date of January 2025. During follow-up, the occurrence of local recurrence or distant metastasis was recorded. Recurrence was defined as the reappearance of tumor at the primary site or adjacent region, confirmed by imaging or pathology. Distant metastasis was defined as the appearance of tumor lesions in organs or tissues beyond the neck, confirmed by imaging or pathology (17). Based on follow-up outcomes, patients were divided into a recurrence/metastasis group and a non-recurrence group. Patients without recurrence or metastasis were followed up until the last follow-up date or the study cutoff date.

### Statistical methods

Data analysis was performed using SPSS 26.0 software. Continuous data were tested for normality. Normally distributed continuous data were presented as mean ± standard deviation and compared via independent samples *t*-tests. Categorical data were presented as numbers and percentages, with comparisons using the *χ²* test or Fisher’s exact test. Pearson’s correlation analysis was used to assess correlations between iron metabolism and oxidative stress indicators. With recurrence or distant metastasis as the outcome event, univariable Cox proportional hazards regression analysis was first performed to screen potential influencing factors. Indicators with *P* < 0.05 were subsequently included in a multivariable Cox regression model to identify independent influencing factors for OSCC recurrence and metastasis. Based on the multivariate results, a predictive model was constructed. Its predictive performance was evaluated using time-dependent receiver operating characteristic (ROC) curves, and the AUC was calculated. A two-sided *P* < 0.05 was considered statistically significant.

## Results

### General patient data and follow-up status

Among the 240 OSCC patients enrolled, there were 154 males and 86 females, with a median age of 55 years (range 28–81 years). The average follow-up time was 46.97 ± 14.03 months (range 11–95 months), with a median of 45 months. During follow-up, 88 patients experienced recurrence and/or metastasis: 50 had local recurrence, 22 had cervical lymph node metastasis (20 ipsilateral, 1 contralateral submandibular, 1 contralateral neck), 8 had distant metastasis (5 lung, 3 esophagus/mediastinum), and 8 had combined recurrence and metastasis (7 cervical lymph node metastasis, 1 multi-site lymph node and bone metastasis). After dividing into recurrence/metastasis and non-recurrence groups, no statistically significant differences were found between the two groups regarding age, gender, smoking history, or alcohol consumption history (all *P* > 0.05; **Table 1**).

**Table 1:** Comparison of general patient data.

Variables	Non-recurrence group (n = 152)	Recurrence/Metastasis group (n = 88)	*t/χ²*	*P*
Age at diagnosis, mean ± SD	55.10 ± 7.97	54.38 ± 8.27	0.669	0.504
Gender, n (%)			0.017	0.896
Female	54 (35.53)	32 (36.36)		
Male	98 (64.47)	56 (63.64)		
Smoking history, n (%)			0.369	0.544
No	77 (50.66)	41 (46.59)		
Yes	75 (49.34)	47 (53.41)		
Alcohol history, n (%)			0.164	0.685
No	84 (55.26)	51 (57.95)		
Yes	68 (44.74)	37 (42.05)		
Hypertension, n (%)			0.481	0.488
No	115 (75.66)	63 (71.59)		
Yes	37 (24.34)	25 (28.41)		
Diabetes, n (%)			0.002	0.966
No	135 (88.82)	78 (88.64)		
Yes	17 (11.18)	10 (11.36)		

Note: SD, Standard deviation.

### Clinicopathological characteristic distribution in recurrence/metastasis patients

Analysis of clinicopathological characteristics showed that the proportion of poorly differentiated tumors was significantly higher in the recurrence/metastasis group compared to the non-recurrence group. T3-T4 stage and N+ status were also more common in this group. Furthermore, incidences of PNI and LVI were significantly higher, and positive margin rates were higher in recurrence/metastasis patients (all *P* ≤ 0.05). These results suggest that adverse clinicopathological features are significantly enriched in OSCC patients with recurrence and metastasis (**Table 2**).

**Table 2:** Distribution of clinicopathological characteristics.

Variables	Non-recurrence group (n = 152)	Recurrence/Metastasis group (n = 88)	*χ²*	*P*
T stage, n (%)			9.263	0.002
T1~T2	106 (69.74)	44 (50.00)		
T3~T4	46 (30.26)	44 (50.00)		
N stage, n (%)			49.127	< 0.001
N0	126 (82.89)	34 (38.64)		
N+	26 (17.11)	54 (61.36)		
Pathological differentiation, n (%)			39.883	< 0.001
Well/Moderate	149 (98.03)	62 (70.45)		
Poor	3 (1.97)	26 (29.55)		
PNI, n (%)			13.691	< 0.001
No	131 (86.18)	58 (65.91)		
Yes	21 (13.82)	30 (34.09)		
LVI, n (%)			8.509	0.004
No	132 (86.84)	63 (71.59)		
Yes	20 (13.16)	25 (28.41)		
Margin status, n (%)			3.835	0.050
Negative	140 (92.11)	73 (83.91)		
Positive	12 (7.89)	14 (16.09)		
Jawbone invasion, n (%)			0.001	1.000
No	146 (96.05)	84 (95.45)		
Yes	6 (3.95)	4 (4.55)		
Primary site, n (%)			9.183	0.102
Buccal mucosa	48 (31.58)	38 (43.18)		
Tongue	40 (26.32)	29 (32.95)		
Gingiva	25 (16.45)	9 (10.23)		
Lip	15 (9.87)	3 (3.41)		
Palate	11 (7.24)	3 (3.41)		
Other sites	13 (8.55)	6 (6.82)		
Treatment regimen, n (%)			0.783	0.854
Surgery alone	78 (51.32)	41 (46.59)		
Surgery + chemotherapy	46 (30.26)	27 (30.69)		
Surgery + radiotherapy	20 (13.16)	13 (14.77)		
Surgery + chemoradiotherapy	8 (5.26)	7 (7.95)		

Note: PNI, Perineural invasion; LVI, Lymphovascular invasion.

### Serum iron metabolism-related indicators between prognostic groups

Patients in the recurrence/metastasis group had significantly higher ferritin levels, while Fe, TF, TIBC, and TSAT were significantly lower (all *P* < 0.05). Overall, this pattern reflects a disordered state of increased iron storage and decreased available iron, suggesting that abnormal iron metabolism may be closely related to OSCC progression and recurrence (**Table 3**).

**Table 3:** Serum iron metabolism-related indicators between prognostic groups.

Variables	Non-recurrence group (n = 152)	Recurrence/Metastasis group (n = 88)	*t*	*P*
Fe (μmol/L), mean ± SD	17.60 ± 4.44	13.90 ± 3.60	6.657	< 0.001
Ferritin (ng/mL), mean ± SD	270.88 ± 61.75	346.70 ± 80.55	-7.627	< 0.001
TF (g/L), mean ± SD	2.33 ± 0.49	2.01 ± 0.36	5.885	< 0.001
TIBC (μmol/L), mean ± SD	58.44 ± 10.07	54.30 ± 7.90	3.532	< 0.001
TSAT (%), mean ± SD	28.60 ± 6.88	22.90 ± 5.70	6.576	< 0.001

Note: Fe, Serum iron; TF, Transferrin; TIBC, Total iron-binding capacity; TSAT, Transferrin saturation; SD, Standard deviation.

### Oxidative stress-related indicators between prognostic groups

Patients in the recurrence/metastasis group had significantly higher MDA and ROS levels but significantly lower SOD and GSH-Px activities compared to the non-recurrence group, and T-AOC also showed a decreasing trend (all *P* < 0.05). These changes indicate significant cumulative oxidative damage and insufficient antioxidant capacity in recurrence/metastasis patients, suggesting that worsened oxidative stress status may contribute to OSCC recurrence or distant dissemination (**Table 4**).

**Table 4:** Oxidative stress-related indicators between prognostic groups.

Variables	Non-recurrence group (n = 152)	Recurrence/Metastasis group (n = 88)	*t*	*P*
MDA (nmol/mL), mean ± SD	3.12 ± 0.68	4.46 ± 1.02	-10.993	< 0.001
ROS (U/mL), mean ± SD	488.90 ± 82.03	512.60 ± 74.55	-2.234	0.027
SOD (U/mL), mean ± SD	146.30 ± 21.63	122.80 ± 19.80	8.360	< 0.001
GSH-Px (U/mL), mean ± SD	77.80 ± 12.56	72.25 ± 14.31	3.130	0.003
T-AOC (mmol/L), mean ± SD	1.36 ± 0.28	1.25 ± 0.25	3.053	0.003

Note: MDA, Malondialdehyde; ROS, Reactive oxygen species; SOD, Superoxide dismutase; GSH-Px, Glutathione peroxidase; T-AOC, Total antioxidant capacity; SD, Standard deviation.

### Correlation analysis between iron metabolism and oxidative stress indicators

**Figure 1:**
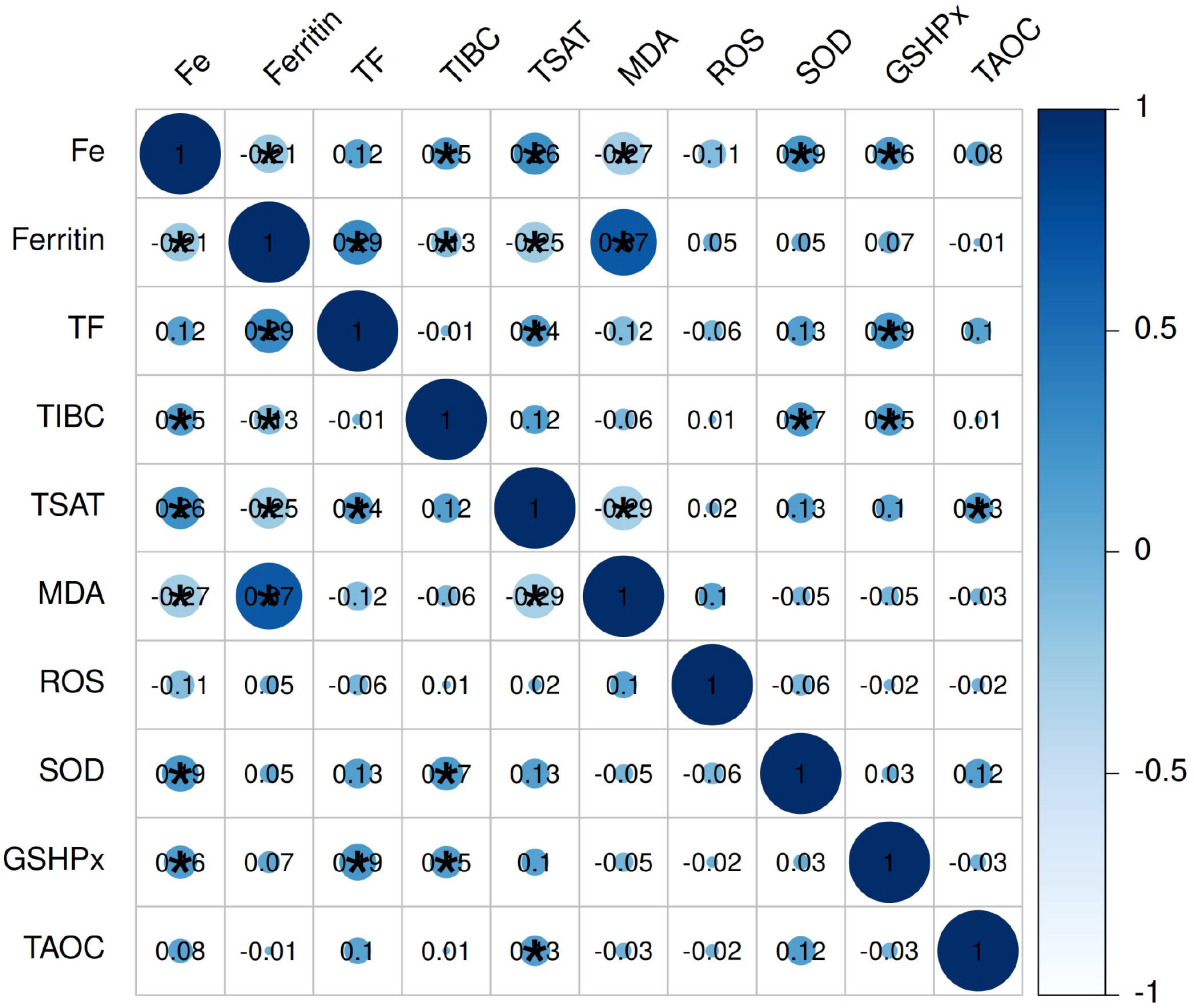
Correlation analysis between iron metabolism and oxidative stress indicators.

Note: * indicates a statistically significant correlation (*P* < 0.05). Fe, Serum iron; TF, Transferrin; TIBC, Total iron-binding capacity; TSAT, Transferrin saturation; MDA, Malondialdehyde; ROS, Reactive oxygen species; SOD, Superoxide dismutase; GSH-Px, Glutathione peroxidase; T-AOC, Total antioxidant capacity.

Further analysis of the relationship between iron metabolism and oxidative stress indicators revealed that ferritin was positively correlated with MDA (*P* < 0.05). Fe was positively correlated with SOD and GSH-Px and negatively correlated with MDA (all *P* < 0.05). TF showed a weak positive correlation with GSH-Px; TIBC correlated weakly with SOD and GSH-Px; and TSAT had a weak positive correlation with T-AOC (all *P* < 0.05). These results suggest a synchronous trend between increased iron load and aggravated oxidative damage, indicating that disruption of iron homeostasis may promote OSCC progression and recurrence by exacerbating oxidative stress (**Figure 1**).

### Cox regression analysis of independent influencing factors for OSCC recurrence/metastasis

In univariable Cox analysis, T stage, N stage, pathological differentiation grade, PNI, LVI, and multiple iron metabolism and oxidative stress-related indicators were significantly associated with OSCC recurrence/metastasis. Compared to T1-T2, T3-T4 patients had a significantly higher risk of recurrence/metastasis (*HR* = 1.723, *95% CI*: 1.134-2.618, *P* = 0.011). N+ patients had a significantly higher risk than N0 patients (*HR* = 3.835, *95% CI*: 2.492-5.901, *P* < 0.001). Patients with poor differentiation had a significantly increased risk (*HR* = 3.764, *95% CI*: 2.375-5.963, *P* < 0.001). Positive PNI (*HR* = 2.007, *95% CI*: 1.290-3.120, *P* = 0.002) and LVI (*HR* = 1.730, *95% CI*: 1.087-2.754, *P* = 0.021) were also significantly associated with higher recurrence/metastasis risk.

Among iron metabolism and oxidative stress indicators, Fe (*HR* = 0.887, *95% CI*: 0.847-0.929, *P* < 0.001), TF (*HR* = 0.230, *95% CI*: 0.135-0.390, *P* < 0.001), TIBC (*HR* = 0.967, *95% CI*: 0.946-0.987, *P* = 0.002), TSAT (*HR* = 0.918, *95% CI*: 0.891-0.946, *P* < 0.001), SOD (*HR* = 0.956, *95% CI*: 0.947-0.965, *P* < 0.001), GSH-Px (*HR* = 0.979, *95% CI*: 0.963-0.996, *P* = 0.016), and T-AOC (*HR* = 0.227, *95% CI*: 0.099-0.519, *P* < 0.001) were protective factors. Ferritin (*HR* = 1.006, *95% CI*: 1.003-1.008, *P* < 0.001) and MDA (*HR* = 1.668, *95% CI*: 1.4320-1.942, *P* < 0.001) were risk factors. Gender, smoking history, alcohol consumption history, age at diagnosis, primary tumor site, and ROS showed no statistically significant association with OSCC recurrence/metastasis (all *P* > 0.05; **Table 5**).

**Table 5:** Univariable Cox regression analysis for OSCC recurrence/metastasis.

Variables	*β*	*S.E*	*Z*	*P*	*HR (95% CI)*
Gender					
Female					1.000 (reference)
Male	0.052	0.222	0.234	0.815	1.053 (0.682-1.627)
Smoking history					
No					1.000 (reference)
Yes	0.043	0.214	0.202	0.840	1.044 (0.687-1.587)
Alcohol history					
No					1.000 (reference)
Yes	-0.127	0.216	-0.586	0.558	0.881 (0.577-1.346)
Hypertension					
No					1.000 (reference)
Yes	0.110	0.236	0.465	0.642	1.116 (0.702-1.774)
Diabetes					
No					1.000 (reference)
Yes	-0.127	0.337	-0.376	0.707	0.881 (0.455-1.705)
T stage					
T1-T2					1.000 (reference)
T3-T4	0.544	0.213	2.551	0.011	1.723 (1.134-2.618)
N stage					
N0					1.000 (reference)
N+	1.344	0.220	6.113	< 0.001	3.835 (2.492-5.901)
Pathological differentiation					
Well/Moderate					1.000 (reference)
Poor	1.325	0.235	5.644	< 0.001	3.764 (2.375-5.963)
PNI					
No					1.000 (reference)
Yes	0.696	0.225	3.092	0.002	2.007 (1.290-3.120)
LVI					
No					1.000 (reference)
Yes	0.548	0.237	2.311	0.021	1.730 (1.087-2.754)
Margin status					
Negative					1.000 (reference)
Positive	0.480	0.292	1.645	0.100	1.616 (0.912-2.863)
Jawbone invasion					
No					1.000 (reference)
Yes	0.440	0.514	0.857	0.392	1.553 (0.567-4.250)
Age at diagnosis	-0.002	0.014	-0.173	0.863	0.998 (0.972-1.024)
Primary site					
Buccal mucosa					1.000 (reference)
Tongue	-0.007	0.247	-0.027	0.979	0.993 (0.613-1.611)
Gingiva	-0.571	0.371	-1.540	0.124	0.565 (0.273-1.169)
Lip	-0.782	0.600	-1.304	0.192	0.457 (0.141-1.482)
Palate	-0.890	0.600	-1.484	0.138	0.410 (0.127-1.330)
Other sites	-0.321	0.440	-0.731	0.465	0.725 (0.306-1.716)
Fe	-0.119	0.024	-5.063	< 0.001	0.887 (0.847-0.929)
Ferritin	0.006	0.001	4.870	< 0.001	1.006 (1.003-1.008)
TF	-1.471	0.270	-5.450	< 0.001	0.230 (0.135-0.390)
TIBC	-0.034	0.011	-3.108	0.002	0.967 (0.946-0.987)
TSAT	-0.085	0.015	-5.560	< 0.001	0.918 (0.891-0.946)
MDA	0.511	0.078	6.577	< 0.001	1.668 (1.432-1.942)
ROS	0.003	0.001	1.868	0.062	1.003 (1.000-1.005)
SOD	-0.045	0.005	-9.483	< 0.001	0.956 (0.947-0.965)
GSH-Px	-0.021	0.009	-2.403	0.016	0.979 (0.963-0.996)
T-AOC	-1.484	0.423	-3.511	< 0.001	0.227 (0.099-0.519)

Note: PNI, Perineural invasion; LVI, Lymphovascular invasion; Fe, Serum iron; TF, Transferrin; TIBC, Total iron-binding capacity; TSAT, Transferrin saturation; MDA, Malondialdehyde; ROS, Reactive oxygen species; SOD, Superoxide dismutase; GSH-Px, Glutathione peroxidase; T-AOC, Total antioxidant capacity; *S.E*: Standard error; *HR*: Hazard ratio; *CI*: Confidence interval.

After including variables with *P* < 0.05 from univariate analysis into the multivariable Cox regression model (with no collinearity detected among indicators, as variance inflation factors were all < 5 and tolerance > 0.1; **Table 6**), the results showed that T stage (*HR* = 2.100, *95% CI*: 1.208-3.650, *P* = 0.009), N stage (*HR* = 2.624, *95% CI*: 1.511-4.555, *P* < 0.001), pathological differentiation grade (*HR* = 1.764, *95% CI*: 1.020-3.051, *P* = 0.042), and some iron metabolism and antioxidant indicators remained independent influencing factors for OSCC recurrence/metastasis. Ferritin (*HR* = 1.010, *95% CI*: 1.001-1.020, *P* = 0.033) was an independent risk factor, while TF (*HR* = 0.182, *95% CI*: 0.092-0.358, *P* < 0.001), TSAT (*HR* = 0.936, *95% CI*: 0.901-0.973, *P* < 0.001), and SOD (*HR* = 0.914, *95% CI*: 0.894-0.934, *P* < 0.001) were independent protective factors. PNI, LVI, Fe, TIBC, MDA, GSH-Px, and T-AOC did not retain statistical significance in the multivariate analysis (all *P* > 0.05; **Table 7**).

**Table 6:** Collinearity diagnostics of variables.

Model		Unstandardized coefficients		Standardized coefficients	*t*	Significance	Collinearity statistics	
		*B*	*S.E.*	*Beta*			Tolerance	VIF
1	Constant	1.743	0.191		9.117	< 0.001		
	T stage	-0.007	0.034	-0.007	-0.195	0.846	0.868	1.153
	N stage	0.134	0.037	0.131	3.643	< 0.001	0.780	1.282
	Pathological differentiation	0.194	0.051	0.131	3.811	< 0.001	0.846	1.183
	PNI	0.048	0.039	0.040	1.223	0.222	0.922	1.085
	LVI	0.007	0.042	0.006	0.167	0.868	0.875	1.143
	Fe	-0.008	0.004	-0.078	-2.205	0.028	0.811	1.233
	Ferritin	0.002	0.000	0.313	6.080	< 0.001	0.380	2.632
	TF	-0.265	0.040	-0.258	-6.627	< 0.001	0.663	1.509
	TIBC	-0.002	0.002	-0.046	-1.397	0.164	0.909	1.100
	TSAT	-0.005	0.002	-0.066	-1.879	0.061	0.811	1.234
	MDA	0.110	0.022	0.238	4.901	< 0.001	0.427	2.344
	SOD	-0.007	0.001	-0.354	-10.263	< 0.001	0.847	1.181
	GSH-Px	-0.003	0.001	-0.096	-2.850	0.005	0.895	1.117
	T-AOC	-0.155	0.058	-0.088	-2.644	0.009	0.917	1.090

Note: *S.E.*, Standard error; VIF, Variance inflation factor; PNI, Perineural invasion; LVI, Lymphovascular invasion; Fe, Serum iron; TF, Transferrin; TIBC, Total iron-binding capacity; TSAT, Transferrin saturation; MDA, Malondialdehyde; SOD, Superoxide dismutase; GSH-Px, Glutathione peroxidase; T-AOC, Total antioxidant capacity.

**Table 7:** Multivariable Cox regression analysis for OSCC recurrence/metastasis.

Variables	*β*	*S.E*	*Z*	*P*	*HR (95% CI)*
T stage					
T1-T2					1.000 (reference)
T3-T4	0.742	0.282	2.628	0.009	2.100 (1.208-3.650)
N stage					
N0					1.000 (reference)
N+	0.965	0.281	3.427	< 0.001	2.624 (1.511-4.555)
Pathological differentiation					
Well/Moderate					1.000 (reference)
Poor	0.568	0.279	2.032	0.042	1.764 (1.020-3.051)
PNI					
No					1.000 (reference)
Yes	0.071	0.256	0.275	0.783	1.073 (0.650-1.772)
LVI					
No					1.000 (reference)
Yes	0.337	0.298	1.131	0.258	1.401 (0.781-2.514)
Fe	-0.050	0.032	-1.580	0.114	0.951 (0.894-1.012)
Ferritin	0.010	0.005	2.126	0.033	1.010 (1.001-1.020)
TF	-1.706	0.347	-4.918	< 0.001	0.182 (0.092-0.358)
TIBC	-0.027	0.017	-1.603	0.109	0.974 (0.943-1.006)
TSAT	-0.066	0.019	-3.386	< 0.001	0.936 (0.901-0.973)
MDA	0.127	0.313	0.405	0.686	1.135 (0.615-2.096)
SOD	-0.090	0.011	-8.042	< 0.001	0.914 (0.894-0.934)
GSH-Px	-0.014	0.010	-1.447	0.148	0.986 (0.967-1.005)
T-AOC	0.384	0.472	0.815	0.415	1.469 (0.583-3.701)

Note: PNI, Perineural invasion; LVI, Lymphovascular invasion; Fe, Serum iron; TF, Transferrin; TIBC, Total iron-binding capacity; TSAT, Transferrin saturation; MDA, Malondialdehyde; SOD, Superoxide dismutase; GSH-Px, Glutathione peroxidase; T-AOC, Total antioxidant capacity; *S.E*: Standard error; *HR*: Hazard ratio; *CI*: Confidence interval.

### Construction of a predictive model for OSCC recurrence/metastasis

**Figure 2:**
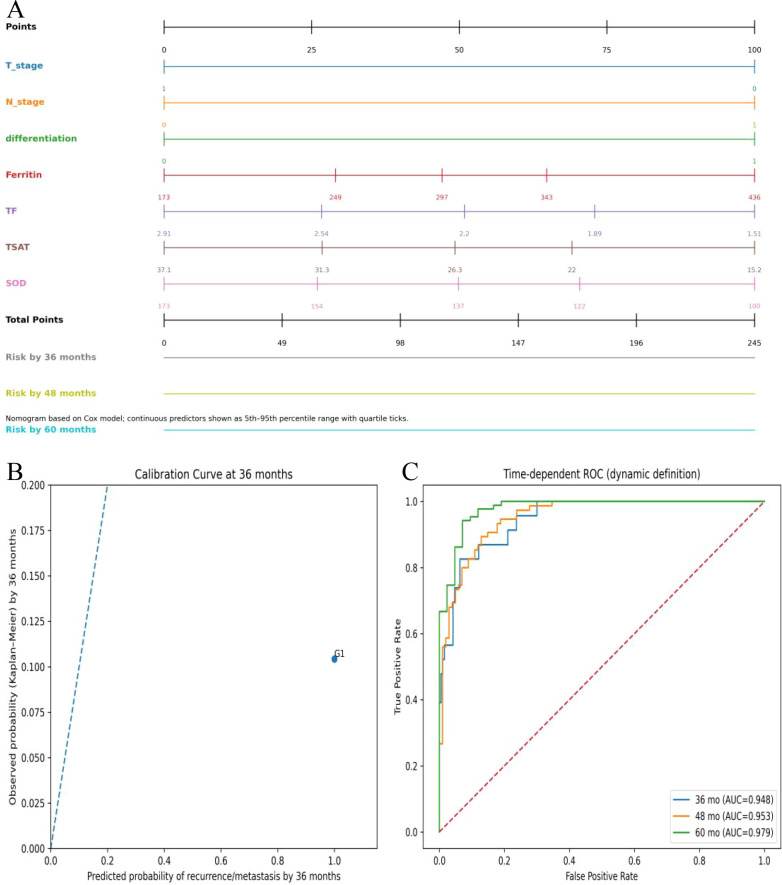
ROC curve of the recurrence/metastasis predictive model based on multivariate analysis. Note: **(A)** Nomogram for predicting the 36-month, 48-month, and 60-month survival probability of OSCC patients. **(B)** Calibration curve at 36 months. **(C)** Time-dependent ROC curves for predicting the 36-month, 48-month, and 60-month survival probability of OSCC patients. ROC, Receiver operating characteristic; OSCC, Oral squamous cell carcinoma; TF, Transferrin; TSAT, Transferrin saturation; SOD, Superoxide dismutase; AUC, Area under the curve.

Based on the results of the multivariable Cox analysis, a predictive model was established using T stage, N stage, differentiation grade, ferritin, TF, TSAT, and SOD. This model was visualized using a nomogram (**Figure 2A**). The model demonstrated good discriminative ability in this cohort (n = 240, recurrence/metastasis events = 88), with a Harrell’s concordance index (C-index) of 0.905. Time-dependent ROC analysis indicated strong discriminatory power of the model at different follow-up time points: the AUCs were 0.948 at 36 months, 0.953 at 48 months, and 0.979 at 60 months (**Figure 2C**). Regarding model calibration, a calibration curve was plotted at the 36-month time point with confidence intervals calculated using bootstrap resampling. The results showed overall agreement between the predicted probabilities and the observed probabilities estimated by the Kaplan-Meier method, suggesting good calibration capability and stability of the model at this time point (**Figure 2B**). These results indicate that the model has excellent performance in predicting survival outcomes for OSCC patients (**Figure 2**).

## Discussion

Based on a single-center retrospective cohort of 240 OSCC patients undergoing radical surgery, with 88 experiencing recurrence and/or metastasis during follow-up, this study highlights that the risk of disease progression remains significant in real-world populations. Regarding clinicopathological factors, the recurrence/metastasis group had a higher proportion of poor differentiation, more frequent T3-T4 stage and N+ status, and higher incidences of PNI, LVI, and positive margins. This confirms that tumor burden, invasive capacity, and local micro-residual disease remain core determinants of OSCC recurrence and metastasis. Therefore, standardized staging evaluation, margin management, and identification of high-risk pathological features remain fundamental to clinical decision-making. This study provides a practical tool for preoperative risk stratification, potentially guiding individualized follow-up and adjuvant therapy decisions.

Building on this, our study provides systematic and quantifiable evidence from the perspective of iron metabolism and oxidative stress. The iron metabolism profile in the recurrence/metastasis group was characterized by elevated ferritin alongside decreased Fe, TF, TIBC, and TSAT, overall consistent with a disordered state of increased iron storage and decreased available iron (18–20). Elevated ferritin and reduced iron availability may directly promote ROS generation, enhancing tumor invasion and metastasis. Tumor-associated inflammation can promote the shift of iron towards storage forms and reduce circulating available iron through the iron homeostasis regulatory axis. Simultaneously, enhanced iron uptake and utilization by tumor cells can drive host changes resembling functional iron deficiency (21, 22). The retained independent effects of TF and TSAT in the multivariate analysis suggest that reduced iron transport capacity or available iron proportion is not merely explained by stage differences but more likely reflects a systemic metabolic-inflammatory state closely linked to tumor progression (23, 24).

Regarding oxidative stress, the recurrence/metastasis group exhibited elevated MDA and ROS levels alongside decreased SOD, GSH-Px and T-AOC, indicating concurrent cumulative lipid peroxidation damage and insufficient antioxidant defense. SOD remained an independent protective factor, highlighting that weakening of the antioxidant barrier is a measurable contributor to recurrence and metastasis (25). In contrast, although MDA showed prominent between-group differences and univariate significance, it lost independence in the multivariate model, implying that it might be a marker resulting from oxidative damage, sharing collinearity or overlapping pathways with stage, iron homeostasis, and antioxidant enzyme activity. More intriguing is the coupling between iron metabolism and oxidative stress: ferritin was positively correlated with MDA, whereas Fe was positively correlated with SOD and GSH-Px and negatively correlated with MDA. This suggests synchronous fluctuations between iron load alterations and oxidative damage levels at the patient level. Mechanistically, iron can amplify oxidative stress by promoting ROS generation and lipid peroxidation, while oxidative stress can further perturb iron homeostasis and remodel the tumor microenvironment. Recent studies indicate that excessive iron and lipid peroxidation can trigger ferroptosis, a form of iron-dependent cell death, which may influence tumor progression and therapy response. OSCC cells may exhibit enhanced iron dependency, rendering them particularly sensitive to disruptions in iron homeostasis and oxidative stress (26–29). This may form a positive feedback loop: disrupted iron homeostasis→increased ROS→oxidative damage→enhanced invasion and metastasis, providing a more coherent metabolic explanatory framework for OSCC recurrence and metastasis (30–33).

In terms of risk prediction, our study integrated T stage, N stage, differentiation grade, ferritin, TF, TSAT, and SOD into a predictive model. Time-dependent ROC analysis revealed AUCs of 0.948 at 36 months, 0.953 at 48 months, and 0.979 at 60 months. These results indicate that combining clinicopathological aggressiveness and systemic iron metabolism/oxidative stress phenotype significantly enhances discriminative ability for recurrence and metastasis risk. The practical significance of this strategy lies in the fact that these serum indicators are obtainable via routine preoperative blood tests. Identifying high-risk individuals preoperatively could provide a more refined basis for decisions on postoperative follow-up frequency, imaging review intensity, neck surveillance, and adjuvant therapy (34). It also offers clinical correlation evidence for exploring potential translational pathways such as modulating iron metabolism, inducing ferroptosis, or intervening in redox homeostasis (35). However, interventions targeting the iron-oxidative stress pathway have a double-edged sword nature. Potential benefits highly depend on the specific population, timing, and tumor biological context, requiring validation through rigorous prospective studies and mechanistic investigations.

This study has several limitations. First, the retrospective single-center design inherently introduces selection and information biases, which may limit the generalizability of our findings. Serum iron and oxidative stress indicators are influenced by systemic conditions such as inflammation, nutritional status, anemia, and liver function, which may act as potential confounders despite adjustment for major oncological variables. Residual confounding may therefore persist. Second, the study relied on single preoperative measurements, which cannot capture dynamic changes in iron metabolism or antioxidant status. Future studies should evaluate longitudinal trends (e.g., change rates in ferritin or SOD) to enhance early prediction of recurrence. Third, although the predictive model showed high discriminative ability within the same cohort, it lacks external validation, raising the possibility of overfitting and limiting immediate clinical applicability. Independent validation in multicenter prospective cohorts is necessary to confirm the model’s generalizability and robustness. Fourth, while recurrence and metastasis outcomes were defined based on clinical and imaging follow-up, specific survival endpoints such as disease-free survival or overall survival were not directly analyzed, which may limit interpretation and comparability with other studies. Fifth, serum indicators reflect systemic status and need corroboration with tumor tissue-level iron homeostasis and oxidative stress phenotypes. Future integration with tissue molecular assays and multi-omics approaches may further elucidate underlying mechanisms and improve the predictive performance of the model.

In summary, this study demonstrates that dysregulated iron metabolism and impaired oxidative stress homeostasis are independently associated with increased risk of recurrence and metastasis in OSCC. Integrating systemic serum biomarkers, including ferritin, TF, TSAT, and SOD, with traditional clinicopathological factors significantly enhances preoperative risk stratification. This approach provides a practical and accessible tool for identifying high-risk patients, guiding individualized follow-up schedules, imaging intensity, and adjuvant therapy decisions. Furthermore, our findings highlight a potential translational pathway, suggesting that targeted modulation of iron metabolism and antioxidant capacity could serve as a novel intervention strategy to mitigate OSCC progression, warranting validation in future prospective and multi-omics studies.

## Data Availability

The original contributions presented in the study are included in the article/supplementary material. Further inquiries can be directed to the corresponding author.
